# Advancements in Biomarkers and Molecular Targets in Hematological Neoplasias

**DOI:** 10.3390/ijms25126570

**Published:** 2024-06-14

**Authors:** Ana Cristina Gonçalves, Raquel Alves, Ana Bela Sarmento-Ribeiro

**Affiliations:** 1 Laboratory of Oncobiology and Hematology (LOH), University Clinics of Hematology and Oncology, Faculty of Medicine (FMUC), University of Coimbra, 3000-548 Coimbra, Portugal; raquel.alves@fmed.uc.pt (R.A.); absarmento@fmed.uc.pt (A.B.S.-R.); 2 Coimbra Institute for Clinical and Biomedical Research (iCBR)—Group of Environmental Genetics of Oncobiology (CIMAGO), FMUC, University of Coimbra, 3000-548 Coimbra, Portugal; 3 Center for Innovative Biomedicine and Biotechnology (CIBB), 3004-504 Coimbra, Portugal; 4 Hematology Service, Centro Hospitalar Universitário de Coimbra, Unidade Local de Saúde de Coimbra, 3000-061 Coimbra, Portugal

Hematological neoplasias are among the most common cancers worldwide, and the number of new cases has been on the rise since 1990, reaching 1.3 million in 2019. However, the age-standardized death rate for all types of hematologic neoplasias has been declining [1]. These diseases are cancers of the hematopoietic and lymphoid tissues arising from the disruption of the development and function of blood cells in bone marrow and/or lymphoid tissues. According to the affected lineage, they are divided into myeloid and lymphoid tumors and classified into several common subtypes, including leukemias, lymphomas, and multiple myeloma [2]. Besides the increased incidence of hematological neoplasias, the mortality rate for all diseases has been declining, reflecting the advances in prevention, early detection, and treatment of these cancers. However, several hematological neoplasms continue to be among the cancers with the highest mortality rates, and relapses and resistance to treatment can occur, reducing the quality of life of patients with these cancers [1]. A better understanding of the molecular mechanisms involved in hematological neoplasias is critical in order to research targets for prevention, treatment, and biomarkers to improve clinical practice.

Advancements in molecular oncology have revealed new insights into the complex mechanisms that govern carcinogenesis, the genomic and proteomic landscape of cancer, cancer metabolism, immune evasion, drug resistance, and relapse, among other crucial areas [3]. This vast amount of knowledge not only helps in the discovery of new molecular targets for treatment but also allows for the identification of essential biomarkers for diagnosing, predicting outcomes, monitoring patients, and assessing the response to therapy. All of these have significant potential for use in clinical practice. Over the last few decades, targeted therapies and cancer biomarkers have significantly improved survival rates and quality of life for people dealing with both hematological and solid tumors [4]. Nowadays, cancer biomarkers encompass diverse biological molecules, including proteins, nucleic acids, and metabolites, sourced from various biological samples such as plasma, circulating tumor cells, extracellular vesicles, and tumor-educated platelets.

The Special Issue, titled “Insights in New Biomarkers and Molecular Drug Targets in Hematological Neoplasias”, aimed to publish current research on the discovery of novel biomarkers and molecular drug targets in hematological malignancies. This collection comprises 11 papers, including four original research articles and seven reviews, with over 65 total citations at present, reflecting the scientific community’s interest in this topic.

Significant advances in clinical medicine have been achieved using biomarkers that allow for a better diagnosis, prognosis, and a more personalized treatment of diseases. These biomarkers centered on cancer patients’ characteristics can be either molecular, cellular, physiologic, or imaging-based. The integration of biomarkers in diagnosis, prognosis, treatment, and disease monitoring has allowed healthcare to move forward regarding more precise and personalized medicine [5]. However, in the case of hematological neoplasias, much more must be accomplished to improve the early and accurate diagnosis of these diseases, to better predict disease outcome and progression, and to customize therapeutic strategies that improve efficacy and reduce side effects. In the context of molecular biomarkers, Bergantim et al. [6] demonstrated the feasibility of isolating and characterizing extracellular vesicles (EVs) obtained from multiple myeloma (MM) patients’ bone marrow and peripheral blood samples at diagnosis and remission. MM is an incurable disease and the third most common hematological neoplasia, accounting for 10% of newly diagnosed hematological neoplasia [7]. This neoplasia arises from the malignant transformation of post-germinal center plasma cells [8]. EVs are a heterogeneous group of lipid bilayer-enclosed particles synthesized and secreted into the extracellular space by different types of cells. Their cargo includes proteins, lipids, and nucleic acids (such as miRNA, mRNA, and DNA) that mediate intercellular communication and contribute to cancer development, progression, and drug resistance transfer. This makes EVs potential sources of biomarkers useful for diagnosis, prognosis, and disease and treatment monitoring for various cancers [9]. In their article, Bergantim and colleagues were able to demonstrate that EVs have the potential to be diagnostic biomarkers, since these EVs at diagnosis present specific multiple myeloma markers, such as CD38 and CD138. Furthermore, EV characterization from paired samples at diagnosis and remission samples showed different molecular signatures, which opens the way to their potential use for measurable residual disease evaluation and the early detection of relapse [6]. Additionally, Ohguchi and Ohguchi [10] summarized the recent knowledge of DIS3 functions in hematopoiesis. They characterized *DIS3* mutations in MM and their clinical impact on disease development and patient prognosis. DIS3 is a ribonuclease that plays a crucial role in immunoglobulin gene rearrangements and B cell maturation, and its loss of function leads to genome instability, contributing to increased chromosomal translocations and mutational burden. In T-cell acute lymphoblastic leukemia (T-ALL), Villa-Morales et al. [11] investigated if NRF2 activation may constitute a prognostic biomarker. They demonstrated that T-ALL patients with high *NFE2L2* expression levels and signal activation showed a significantly lower disease-free survival rate compared to those with low *NFE2L2* expression and signaling. The *NFE2L2* gene encodes the transcription factor NRF2 (nuclear factor erythroid 2-related factor 2), which plays a key role in protecting the cell against oxidative and electrophilic stress. This transcription factor plays a dual role in cancer, serving as a tumor suppressor and promoter. NRF2 prevents cancer development by protecting cells from oxidative stress and damage. However, after cancer development, persistent NRF2 activation supports tumor growth and survival by boosting cellular defenses and promoting resistance to chemotherapy [12]. The article of Villa-Morales et al. analyzed a publicly available T-cell acute lymphoblastic leukemia gene expression database that revealed a correlation between *NFE2L2* expression and the TLX3 molecular subgroup of T-ALL, which was already associated with poor outcomes. Altogether, these results strongly support the notion that high *NFE2L2* expression indicates poor prognosis in T-ALL and may constitute a new prognostic biomarker for patient risk stratification [11]. 

The biomarker potential of NFE2L2 was already described for other cancer types, including low-grade glioma [13], head and neck squamous cell cancer [14], and colorectal cancer [15]. Moreover, the NRF2 pathway already showed its potential as a molecular target for acute lymphoblastic leukemia [16]. However, cancer involves several cellular and molecular mechanisms, and gene transcription and mRNA translation have gained more interest in cancer research as potential biomarkers and targets for therapy. These processes are tightly regulated by RNA-binding proteins (RBPs), a diverse class of proteins that interact with transcripts and noncoding RNAs, influencing blood cell differentiation and contributing to hematological cancer development and progression. The role and potential clinical significance of RNA-binding proteins (RBPs) in hematological malignancies were discussed by Aguilar-Garrido et al. [17]. They highlighted the importance of understanding the regulation of RBPs, which act as master switches in cancer, aging, and hematopoiesis. Additionally, they discussed the importance of understanding its dysregulation in hematological malignancies to identify new molecular targets and potential disease biomarkers. 

With a focus on molecular targets and new therapy strategies in hematological malignancies, Gallazzi et al. [18] describe the major molecular targets of immunotherapy and explain the mode of action of novel monoclonal antibody (mAb)-based immunotherapies for acute myeloid leukemia (AML) and myelodysplastic neoplasias (MDS). The authors highlighted the main results from several clinical trials testing innovative mAbs in AML and MDS. On the other hand, Van Laethem et al. examined the role of leukocyte-associated immunoglobulin (Ig)-like receptor 1 (LAIR1), a receptor belonging to the immune-inhibitory receptor family, in normal physiological conditions and in hematological neoplasias. Their paper discusses potential therapeutic strategies targeting LAIR1, which is widely expressed in hematopoietic mature cells, for treating various autoimmune diseases and hematological neoplasias [19]. Furthermore, Maher and colleagues [20] reviewed and discussed several biomarkers of refractoriness to chemoimmunotherapy and inhibitors of Bruton tyrosine kinase (BTK) and BCL2 in chronic lymphocytic leukemia. Since several novel immunotherapy agents are under investigation for the treatment of chronic lymphocytic leukemia in patients, these authors also discussed potential biomarkers of refractoriness to immunotherapy in these patients.

Additionally, Al-Odat and colleagues [21] highlighted the molecular processes underlying the interaction between apoptosis and autophagy. These two biological processes are sources of molecular targets for therapeutic applications, and the crosslink between them plays a significant role in MM progression and drug resistance. In this review, the authors also summarize the studies that evaluated the impact of co-targeting apoptosis and autophagy to treat MM. Since pre-clinical models are of great importance to understanding the biology of MM and studying new therapeutic strategies, Lourenço et al. [22] highlighted the role of bone marrow microenvironment cells in MM and how current therapeutic drugs can target them. They also discussed the strengths and limitations of the existing patient-derived 3D MM models and their potential to identify MM molecular mechanisms and new therapeutic targets. These authors concluded that the best model to study MM should be based on patient samples since every tumor has a unique biology. 

Patients with hematological neoplasias often face the challenge of developing resistance to existing therapies, making their treatment a complex task. However, the exploration of new therapeutic strategies can open up alternative options for patients who do not respond to current treatments, thereby improving survival rates and quality of life. This Special Issue also includes papers that explore new therapeutic options in acute leukemias. Greilberger et al. [23] examined the synergistic effect of alpha-ketoglutarate (aKG) and 5-hydroxymethyl-furfural (5-HMF) on T-cell acute lymphoblastic leukemia. Their experiments suggested that 5-HMF has a cytostatic effect, and the combination treatment of this compound with aKG showed a solid antitumoral effect with the activation of apoptosis. This combination reduced oxidative modifications and increased antioxidative capacity, leading to apoptosis. Finally, Jorge and colleagues [24] demonstrated that parthenolide, a naturally occurring sesquiterpene lactone, significantly induced apoptosis through oxidative stress, with increased reactive oxygen species and decreased glutathione levels, in B- and T-lymphoid leukemias. They also observed that apoptosis induction was associated with lower mitochondrial membrane potential, increased activated caspase-3, FAS-ligand expression, and decreased NF-κB phosphorylation. 

This compilation of original research articles and reviews provides an overview of some of the latest advancements in comprehending molecular mechanisms, emerging molecular biomarkers, and potential drug targets in hematological neoplasias. We expect that these breakthroughs will spark enthusiasm among fundamental and clinical cancer researchers working to improve patients’ approaches to hematological neoplasias.

## Data Availability

All data generated or analyzed during this study are included in this published article.
